# Quantitative Proteomics Study of Larval Settlement in the Barnacle *Balanus amphitrite*

**DOI:** 10.1371/journal.pone.0088744

**Published:** 2014-02-13

**Authors:** Zhang-Fan Chen, Huoming Zhang, Hao Wang, Kiyotaka Matsumura, Yue Him Wong, Timothy Ravasi, Pei-Yuan Qian

**Affiliations:** 1 KAUST Global Collaborative Research Program, Division of Life Science, The Hong Kong University of Science and Technology, Hong Kong SAR, China; 2 Bioscience Core Laboratory, King Abdullah University of Science and Technology, Thuwal, Kingdom of Saudi Arabia; 3 Integrative Systems Biology Lab, King Abdullah University of Science and Technology, Thuwal, Kingdom of Saudi Arabia; National Center for Biotechnology Information (NCBI), United States of America

## Abstract

Barnacles are major sessile components of the intertidal areas worldwide, and also one of the most dominant fouling organisms in fouling communities. Larval settlement has a crucial ecological effect not only on the distribution of the barnacle population but also intertidal community structures. However, the molecular mechanisms involved in the transition process from the larval to the juvenile stage remain largely unclear. In this study, we carried out comparative proteomic profiles of stage II nauplii, stage VI nauplii, cyprids, and juveniles of the barnacle *Balanus amphitrite* using label-free quantitative proteomics, followed by the measurement of the gene expression levels of candidate proteins. More than 700 proteins were identified at each stage; 80 were significantly up-regulated in cyprids and 95 in juveniles vs other stages. Specifically, proteins involved in energy and metabolism, the nervous system and signal transduction were significantly up-regulated in cyprids, whereas proteins involved in cytoskeletal remodeling, transcription and translation, cell proliferation and differentiation, and biomineralization were up-regulated in juveniles, consistent with changes associated with larval metamorphosis and tissue remodeling in juveniles. These findings provided molecular evidence for the morphological, physiological and biological changes that occur during the transition process from the larval to the juvenile stages in *B. amphitrite*.

## Introduction

The life cycles of most sessile marine invertebrates include a microscopic and planktonic larval stage that may last for minutes to months, as well as benthic juvenile and adult stages during which individuals attach to a submerged surface [1]. The transition from pelagic to sessile stages is referred to as larval attachment and metamorphosis (collectively known as larval settlement), which is associated with morphological, physiological and biochemical changes. Larval settlement is crucial not only for recruitment but also for species distribution and community structures [2]. Although details of the signal transduction pathways and mechanisms that regulate larval settlement have been partially reported in some species [3], [4], the associated molecular mechanisms remain largely unknown in most marine invertebrate species, due to their high biological diversity.

The barnacle *Balanus amphitrite* is a dominant fouling organism worldwide. *B. amphitrite* larvae released from adults molt 6 times and transit to cyprids, the competent stage for subsequent settlement. The process of settlement can be divided into 3 phases: attainment of competency, attachment to a suitable substratum, and metamorphosis into juveniles [5]. The morphogenetic development associated with metamorphosis includes decortication of the cyprid carapace, formation of a new chitinous layer, migration of the naupliar eye, degeneration of the compound eyes and antenna, and development of the feeding cirri [6]. In addition, physiological, structural and functional changes occur, all of which are regulated by functional genes and proteins [7]. Six cyprid-specific genes were first isolated from a cyprid cDNA library [8], and responded differentially to settlement cues [9]. Recently, we conducted a comparative transcriptomic study and identified several genes with potential roles in the larval settlement process [5].

There is no predictive correlation between mRNA and protein levels. Because proteins directly mediate most biological events, evaluation of changes in their levels could provide comprehensive biological insights [10]. An earlier 2-DE-based proteomic study from our laboratory revealed approximately 400 spots and identified some proteins that were differentially expressed during barnacle larval settlement [10]. Furthermore, a significantly higher number of protein spots were obtained when implementing additional solution-phase IEF sample prefractionation and narrow-pH-range IEF [11]. However, the 2-DE method has a relatively poor reproducibility, low sensitivity, and narrow linear dynamic ranges [12]. In addition, few proteins exhibiting a relatively lower expression level could be identified using mass spectrometry in a 2-DE-based analysis. In contrast, a gel-free proteomics technique incorporating a combination of multidimensional liquid chromatography (LC) separation, MS analysis and sequence database searches could provide a robust and effective platform for direct analysis of the proteome of the bryozoan *Bugula neritina* [13]. In the present study, we used a label-free quantitative proteomic platform to profile the proteomes of 4 developmental stages of *B. amphitrite*, compared 4 proteomes to identify many differentially expressed proteins that might play key roles in the settlement of *B. amphitrite*, and confirmed the expression patterns of numerous proteins using quantitative real-time polymer chain reaction (qRT-PCR).

## Materials and Methods

### Sample preparation

The barnacle, *Balanus amphitrite*, that we used for this study is a common species of marine invertebrates. It is a biofouling species and not endangered or protected. *Balanus amphitrite* adults were collected from a dock in Pak Sha Wan, Hong Kong (22.21′45″ N, 114.15′35″ E). No specific permits were required for the described field studies. The dock does not belong to any national parks, protected areas, or privately-owned places. The filed studies did not involve any endangered or protected species.

Larvae of different stages were obtained and cultured according to the methods described by Thiyagarajan and Qian [10]. Briefly, newly released larvae were maintained in filtered seawater (FSW) for 2 h and then collected as stage II nauplii. Other larvae were cultured at 27°C and fed with *Chaetoceros gracilis* Schutt for 3 to 4 d until they had developed into stage VI nauplii with 2 compound eyes. After 18–24 h, a portion of the cyprids undergoing molting from stage VI nauplii was collected; the remaining cyprids attached to polystyrene Petri dishes (Falcon no. 1006) in the dark. Most of the cyprids attached to the dishes within 24 h and completed metamorphosis into juveniles within 48 h. The juveniles were then scraped off the dishes. All of the samples were stored at −80°C until use.

### Protein extraction and digestion

The samples were resuspended in 1 mL protein lysis buffer containing 0.1 M Tris-HCl (pH 7.6), 2% SDS, 0.1 M dithiothreitol and protease inhibitor cocktail (Roche Diagnostics, Mannheim, Germany). After homogenization, the samples were sonicated 3 times using a Misonix sonicator-XL2020 (Misonix, Farmingdale, NY) and then incubated in boiling water for 5 min. Larvae and debris that were not lysed were pelleted by centrifugation at 15,000 g for 10 min at 4°C. The protein-containing supernatant was transferred into a new tube, and the protein concentration was quantified using the RC/DC protein assay kit (BioRad, Hercules, CA). Due to the presence of pigments and other contaminants in the barnacle protein extract, one dimensional gel electrophoresis was performed before trypsin digestion to remove impurities, including pigments, detergents, and buffer components. This purification step facilitated the subsequent liquid chromatographic separation. Specifically, protein samples were loaded in a 10% SDS-PAGE gel and separated for 25 min to purify the proteins from other non-proteins/small molecules. Following Coomassie blue staining, the concentrated protein bands were removed from the gel and ready for in-gel digestion as previously described [14].

### Liquid chromatography/mass spectrometry (LC/MS) analysis

The digests were resuspended and fractionated by strong cation exchange chromatography [15]. Each dried fraction was reconstituted in 30 μL of 0.1% formic acid. The samples were run as 3 replicates using a Proxeon EASY-nLC unit (Thermo Scientific, San Jose, USA) with an LTQ-Orbitrap mass spectrometer (Velos, Thermo Scientific). Peptide separation was conducted in a capillary column (0.1×150 mm, with C18 AQ of 3 μm particles and 200 Å pore size, Bruker Michrom BioResources). Mobile phase A (0.1% formic acid in H_2_O) and mobile phase B (0.1% formic acid in ACN) were used to establish a 75-min gradient consisting of 45 min from 100 to 65% A, 10 min from 65 to 20% A, and 20 min at 20% A. The LC was operated at a constant flow rate of 0.5 μL/min. The ion source was set as a capillary voltage of 1.5 kV and a source temperature of 160°C. The LTQ-Orbitrap was set to perform data-dependent acquisition in positive ion mode with a selected MS survey mass range of 350-1600 *m*/*z*. The 10 most intense ions above a 500-count threshold and carrying a charge from 2+ to 4+, were selected for MS/MS fragmentation. Dynamic exclusion was activated using a repeat count of 2, an exclusion duration of 45 s, and a mass tolerance of ±5 ppm. The CID parameters included a normalized collision energy of 35%, an activation Q of 0.25, an isolation width of 3.0 and an activation time for 10 ms.

### Database search and data analysis

The raw MS data were converted into mascot generic files using Proteome Discover (1.2) and then submitted to Mascot version 2.2 (Matrix Sciences Ltd., London, UK) for searching against an in-house protein database developed in our transcriptome study [5]. The mass tolerances were set at 10 ppm for the peptide precursors and 0.5 Da for the fragment ions. A decoy option was included. Carboxamidomethylation at cysteine residues was set as a fixed modification, and oxidation at methionine residues was set as a variable modification. Up to 1 missed trypsin cleavage was permitted.

The resulting .dat files from the Mascot search were processed using Scaffold (version 4.0, Proteome Software Inc., OR, USA) to validate the MS/MS identification. Peptide identification was accepted if the result could established at >95.0% probability by the Scaffold Local FDR, whereas protein identification was accepted if the result had a probability of >99.0% and contained at least 1 identified peptide. Peptide and protein probabilities assigned by the PeptideProphet [16] and ProteinProphet [17]. Protein XML files were then exported from Scaffold to calculate the protein abundances were calculated using APEX quantitation proteomics tools [18], [19]. A 1.5-fold change was set as the cutoff, and only proteins with >5,000 molecules per cell during at least one stage were considered for significantly up-regulated proteins and proteins with >5,000 molecules per cell during at least 3 stages were considered for significantly down-regulated proteins [20].

### Phylogenetic analysis of vitellogenin

Amino acid sequences of vitellogenin from various species were downloaded from GenBank and aligned using MUSCLE [21]via the CIPRES Portal v2.2 [22]. Neighbor-joining analysis was performed with MEGA4. All of the positions containing alignment gaps and missing data were eliminated in pairwise sequence comparisons (pairwise deletion option). The topological stability was evaluated based on 1,000 bootstrapping (BS) replications.

### Quantitative real-time polymerase chain reaction (qRT-PCR)

Total RNA from each stage as well as from adults was extracted by using TRIzol reagent (Invitrogen, Carlsbad, CA) according to the manufacturer's protocol. The quantity and quality of the RNA were assessed using agarose gel electrophoresis and a NanoDrop 1000 spectrophotometer (Thermo Fisher Scientific, Waltham, MA), respectively. Trace genomic DNA in the RNA solution was removed using the Turbo DNAfree Kit (Ambion Inc, Austin, TX). First-strand cDNA was synthesized from RNA using MMLV reverse transcriptase (USB, Cleveland, OH) with oligo dT_(18)_ primer. Gene-specific primers were designed with Primer3 software [23], and the sequences of all primers used are listed in Table S1. According to the standard protocol, the qRT-PCR assays were conducted using SYBR Green Supermix (BioRad) on an ABI 7500 fast real-time PCR machine (Applied Biosystems, Foster City, CA). Cytochrome b (*Cyb*) was employed as an internal control for normalization [24]; the relative expression patterns were calculated based on the 2^−ΔΔCt^ method [25], [26]. Significant differences in expression patterns were analyzed by one-way ANOVA followed by the Tukey's post-hoc test.

## Results

**Figure 1:**
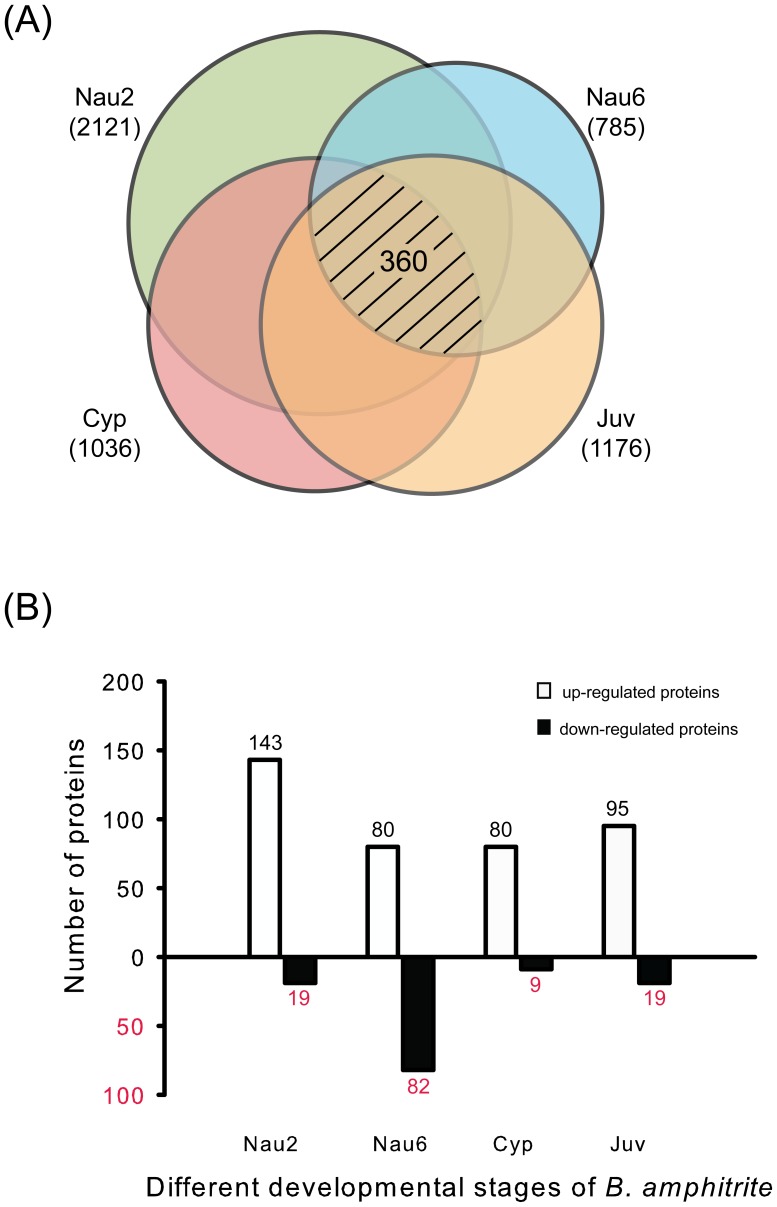
The numbers of overlap proteins among the 4 developmental stages of *B. amphitrite* (A) and the numbers of differentially expressed proteins during each developmental stage (B). In the Venn diagram, the numbers in parentheses are the number of quantified proteins expressed during each stage. Shaded numbers show the number of the common proteins; Nau2: stage II nauplii, Nau6: stage VI nauplii, Cyp: cyprids, and Juv: juveniles.

**Figure 2:**
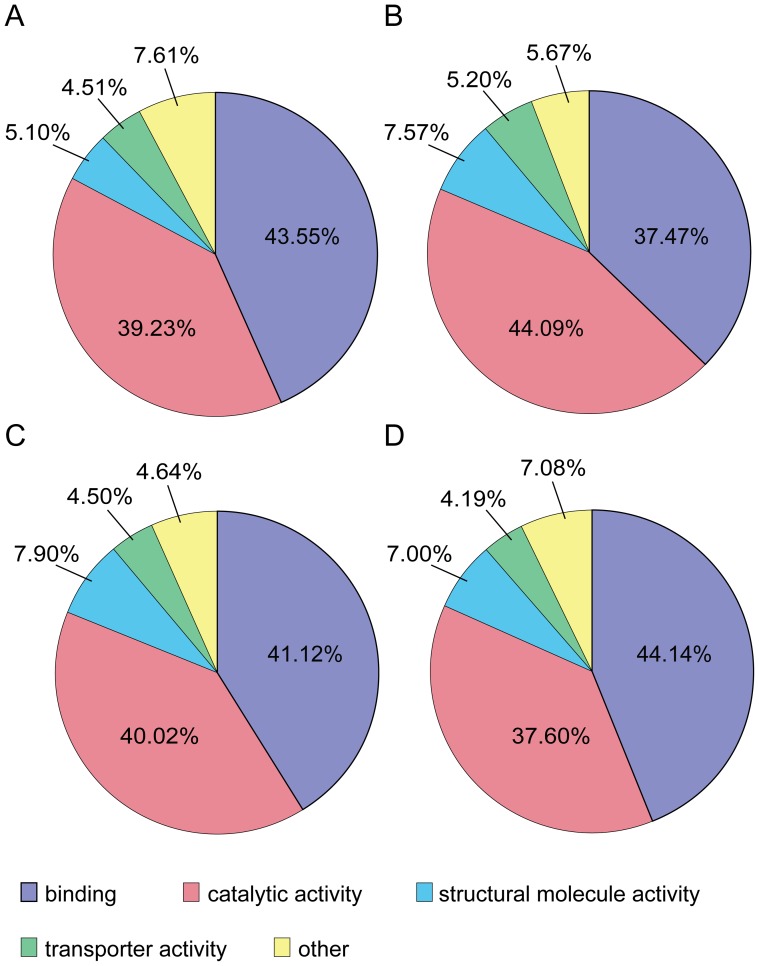
Percentage distribution of the molecular functions of identified proteins in 4 developmental stages, including stage II nauplii (A), stage VI nauplii (B), cyprids (C) and juveniles (D). The top 4 categories were binding, catalytic activity, structural molecule activity and transporter activity, followed by regulators of the activity of enzymes, receptors, molecular transducers, electron carriers, nucleic acid binding transcription factors, protein binding transcription factors, antioxidants, translation regulators, metallochaperones, and receptor regulators.

Four developmental stages of *B. amphitrite* were collected for our proteomic profiling. After MS profiling, 2,121 proteins were isolated from stage II nauplii, 785 from stage VI nauplii, 1,036 from cyprids, and 1176 from juveniles (Figure 1A, Table S2). Among the total 2,520 proteins, 360 proteins were common to all 4 developmental stages. Using gene ontology (GO), 1,793 of 2,520 proteins (accounting for 71.15% of the total proteins) were categorized into several functional groups, including 1,555 of 2,118 (73.42%) in stage II nauplii, 570 of 783 (72.80%) in stage VI nauplii, 790 of 1,034 (76.40%) in cyprids and 895 of 1,174 (76.24%) in juveniles (GO categories are shown in Figure 2). The majority of the proteins displayed binding and catalytic activity (37.47%–44.14%), and the remainder were associated with structural molecule activity and transporter functions (4.19%–7.90%). Unlike the other 3 developmental stages, stage VI nauplii had more proteins that displayed catalytic (44.09%) rather than with binding activity (37.47%). The remaining categories included electron carrier activity, enzyme regulator activity, receptor activity, antioxidant activity, nucleic acid binding, and transcription factor activity, among others.

Comparative proteomic analysis among the 4 developmental stages of *B. amphitrite* revealed that 398 proteins met the established criteria, displaying a 1.5-fold change and >5,000 molecules per cell in at least one stage (Figure 1B). Among them, 143 in stage II nauplii, 80 in stage VI nauplii, 80 in cyprids and 95 in juveniles were significantly up-regulated (listed in Tables 1, 2, S3 and S4). Interestingly, 118 proteins were uniquely expressed in stage II nauplii, accounting for 82.51% of the total 143 proteins. In contrast, 36, 22 and 27 proteins were uniquely expressed in stage VI nauplii, cyprids and juveniles, respectively. In addition, 129 proteins were significantly down-regulated in the 4 developmental stages, which is less than the up-regulated ones (Figure 1B). Among them, 19 were in stage II nauplii, 82 in stage VI nauplii, 9 in cyprids and 19 in juveniles (listed in Table S5, S6, S7 and S8). To identify the important proteins involved in the larval settlement process of barnacles, proteins that were significantly up-regulated in cyprids (listed in Table 1) were categorized into a diverse set of functional groups using GO and eukaryotic orthologous groups (KOG). Approximately 30% proteins were related to energy and metabolism; others were associated with structural molecules, nervous system-related molecules, and signaling molecules, among others. Additionally, proteins that were significantly up-regulated in juveniles (Table 2) might be crucial for tissue reorganization and development postlarval settlement. The top 3 categories detected were structural, transcription and translation, and energy and metabolism proteins. The other proteins were categorized into various functional groups such as cell differentiation-related, cell proliferation-related, shell calcification-related, and stress-induced proteins.

**Table 1:** Significantly Up-regulated Proteins in Cyprids^a^.

		Protein abundance (×1000 molecules/cell)								Protein ratios								
		nau2		nau6		cyp		juv		nau2/cyp			nau6/cyp			juv/cyp		
Accession No.	Protein Description	ave	SD	ave	SD	ave	SD	ave	SD	ave	SD	*p*	ave	SD	*p*	ave	SD	*p*
**Energy and Metabolism**																		
CL5556.Contig1	Chromosome 14 open reading frame 149	N.D.	N.D.	8.78	2.01	29.75	0.93	N.D.	N.D.	0.00	0.00	0.000	0.30	0.07	0.001	0.00	0.00	0.000
Unigene12197	Serine hydroxymethyl-transferase, mitochondria	4.03	2.33	N.D.	N.D.	16.26	2.86	N.D.	N.D.	0.25	0.14	0.005	0.00	0.00	0.002	0.00	0.00	0.000
CL13389.Contig1	prolyl endopeptidase	2.59	0.90	1.54	0.07	21.40	1.52	6.53	0.62	0.12	0.04	0.000	0.07	0.00	0.000	0.30	0.03	0.000
CL6434.Contig1	Cytosolic non-specific dipeptidase	16.25	1.39	23.57	6.75	47.51	3.40	26.53	2.30	0.34	0.03	0.000	0.50	0.14	0.015	0.56	0.05	0.001
Unigene15104	CG3999 CG3999-PA	N.D.	N.D.	N.D.	N.D.	23.99	5.36	N.D.	N.D.	only in cyp		0.004	only in cyp		0.004	only in cyp		0.004
Unigene6569	dimethylglycine dehydrogenase, mitochondrial-like	N.D.	N.D.	N.D.	N.D.	8.65	2.53	N.D.	N.D.	only in cyp		0.007	only in cyp		0.007	only in cyp		0.007
CL346.Contig4	4-aminobutyrate aminotransferase	N.D.	N.D.	N.D.	N.D.	10.55	5.06	N.D.	N.D.	only in cyp		0.020	only in cyp		0.020	only in cyp		0.020
CL10509.Contig1	betaine homocysteine methyl transferase	5.65	0.45	N.D.	N.D.	13.64	1.93	N.D.	N.D.	0.41	0.03	0.001	0.08	0.00	0.000	0.00	0.00	0.002
CL8097.Contig1	betaine homocysteine methyltransferase	N.D.	N.D.	N.D.	N.D.	10.79	2.59	N.D.	N.D.	only in cyp		0.005	only in cyp		0.005	only in cyp		0.005
CL4185.Contig1	mannose-6-phosphate isomerase	3.34	0.11	N.D.	N.D.	6.14	0.29	0.00	0.00	0.54	0.02	0.000	0.00	0.00	0.000	0.00	0.00	0.000
CL16245.Contig1	fructose 1,6-bisphosphatase	9.63	0.67	11.37	2.59	62.13	2.63	18.61	3.70	0.16	0.01	0.000	0.18	0.04	0.000	0.30	0.06	0.000
CL288.Contig4	fructose-biphosphate aldolase	40.69	2.27	13.01	3.27	108.11	10.44	66.50	5.09	0.38	0.02	0.000	0.12	0.03	0.001	0.62	0.05	0.003
CL1136.Contig1	glucose-6-phosphate isomerase	13.97	3.48	N.D.	N.D.	31.90	3.55	18.55	2.05	0.44	0.11	0.004	0.00	0.00	0.001	0.58	0.06	0.004
CL280.Contig1	CG11255 CG11255-PB	10.29	3.39	N.D.	N.D.	44.27	2.72	16.98	7.27	0.23	0.08	0.001	0.00	0.00	0.000	0.38	0.16	0.007
CL9352.Contig1	glucosamine-6-phosphate isomerase 1	3.33	1.28	2.06	0.02	19.44	2.63	10.73	4.13	0.17	0.07	0.001	0.11	0.00	0.003	0.55	0.21	0.040
CL1148.Contig1	neutral alpha-glucosidase AB	8.25	1.89	N.D.	N.D.	17.70	2.65	7.53	1.70	0.47	0.11	0.007	0.00	0.00	0.002	0.43	0.10	0.005
CL3803.Contig1	Acetyl-CoA acetyltransferase, mitochondrial	10.95	0.56	N.D.	N.D.	19.65	3.45	6.09	2.61	0.56	0.03	0.008	0.00	0.00	0.002	0.31	0.13	0.007
CL4830.Contig1	Acad8 protein	N.D.	N.D.	N.D.	N.D.	12.29	3.92	N.D.	N.D.	only in cyp		0.009	only in cyp		0.009	only in cyp		0.009
CL10791.Contig1	acyl-CoA oxidase	21.55	1.27	5.72	0.21	46.67	0.50	29.83	3.10	0.46	0.03	0.000	0.12	0.00	0.000	0.64	0.07	0.013
Unigene30254	acetyl-CoA carboxylase 2	6.81	2.06	N.D.	N.D.	18.31	0.73	N.D.	N.D.	0.37	0.11	0.002	0.00	0.00	0.000	0.00	0.00	0.000
Unigene4811	uridine 5′-monophosphate synthas	N.D.	N.D.	N.D.	N.D.	21.71	11.80	N.D.	N.D.	only in cyp		0.026	only in cyp		0.026	only in cyp		0.026
CL1575.Contig1	aldehyde dehydrogenase 1 family, member L1	7.02	0.63	N.D.	N.D.	21.42	3.69	6.15	3.29	0.33	0.03	0.001	0.00	0.00	0.003	0.29	0.15	0.011
CL1120.Contig2	pyrroline-5-carboxylate dehydrogenase	20.82	0.76	26.34	6.95	69.73	3.99	28.92	3.05	0.30	0.01	0.000	0.38	0.10	0.004	0.41	0.04	0.000
CL6520.Contig1	hydroxyacid-oxoacid transhydrogenase, mitochondrial	5.39	2.42	N.D.	N.D.	20.87	1.39	N.D.	N.D.	0.26	0.12	0.002	0.00	0.00	0.000	0.00	0.00	0.000
CL1939.Contig1	malate synthase, glyoxysomal	N.D.	N.D.	N.D.	N.D.	14.20	3.58	4.67	1.18	0.00	0.00	0.005	0.00	0.00	0.005	0.33	0.08	0.007
CL5668.Contig1	CG7280 CG7280-PA	1.62	0.69	N.D.	N.D.	17.34	4.89	4.95	4.05	0.09	0.04	0.001	0.07	0.00	0.006	0.29	0.23	0.028
CL271.Contig3	mitochondrial aldehyde dehydrogenase precursor	27.10	1.68	N.D.	N.D.	63.61	5.66	32.13	5.27	0.43	0.03	0.000	0.00	0.00	0.001	0.51	0.08	0.003
**Structural Molecules**																		
Unigene13912	projectin	6.34	0.39	8.77	0.11	23.62	1.19	6.36	0.53	0.27	0.02	0.000	0.37	0.00	0.000	0.27	0.02	0.000
Unigene28824	Titin	6.83	0.69	N.D.	N.D.	14.87	0.34	N.D.	N.D.	0.46	0.05	0.000	0.00	0.00	0.000	0.00	0.00	0.000
CL682.Contig1	Muscle M-line assembly protein unc-89	1.11	0.39	N.D.	N.D.	6.62	1.26	N.D.	N.D.	0.17	0.06	0.001	0.00	0.00	0.003	0.00	0.00	0.000
Unigene3292	translocase of outer membrane 34	N.D.	N.D.	N.D.	N.D.	5.96	0.34	N.D.	N.D.	only in cyp		0.000	only in cyp		0.000	only in cyp		0.000
Unigene5637	twitchin	3.99	0.15	N.D.	N.D.	20.24	1.31	N.D.	N.D.	0.20	0.01	0.000	0.00	0.00	0.000	0.00	0.00	0.000
Unigene10292	Titin	7.42	2.30	8.04	0.19	16.36	2.76	6.73	2.09	0.45	0.14	0.012	0.49	0.01	0.018	0.41	0.13	0.009
Unigene2708	BMKETTIN	3.60	3.14	N.D.	N.D.	21.33	6.94	N.D.	N.D.	0.17	0.15	0.029	0.00	0.00	0.010	0.00	0.00	0.010
CL1129.Contig1	Echinoderm microtubule-associated protein	4.68	0.86	N.D.	N.D.	11.25	2.12	2.50	2.20	0.42	0.08	0.005	0.00	0.00	0.003	0.22	0.20	0.037
**Transcription and Translation**																		
CL128.Contig1	ribosomal protein L10	16.26	4.07	5.93	1.62	29.64	1.00	18.58	1.00	0.55	0.14	0.009	0.20	0.05	0.001	0.63	0.03	0.000
CL136.Contig1	ribosomal protein L18A family member	5.59	1.02	11.50	1.33	20.06	1.63	N.D.	N.D.	0.28	0.05	0.000	0.57	0.07	0.008	0.00	0.00	0.001
CL814.Contig1	40S ribosomal protein S2	9.17	1.01	7.44	1.78	25.55	2.99	15.14	1.26	0.36	0.04	0.001	0.29	0.07	0.004	0.59	0.05	0.004
Unigene11657	ribosomal protein L9e	15.02	3.74	16.52	1.52	42.90	2.09	23.35	7.07	0.35	0.09	0.001	0.39	0.04	0.001	0.54	0.16	0.014
CL6677.Contig1	translational activator GCN1	12.85	1.02	N.D.	N.D.	21.49	1.69	11.36	3.60	0.60	0.05	0.001	0.00	0.00	0.001	0.53	0.17	0.019
CL2773.Contig1	10-formyltetrahydrofolate dehydrogenase	1.59	0.13	N.D.	N.D.	11.22	2.23	N.D.	N.D.	0.14	0.01	0.008	0.00	0.00	0.003	0.00	0.00	0.003
**Stressed-Induced Protein**																		
CL1841.Contig1	catalase	10.20	1.92	N.D.	N.D.	27.14	2.84	5.48	1.22	0.38	0.07	0.001	0.00	0.00	0.001	0.20	0.05	0.000
CL7722.Contig1	glutathione S-transferase mu	3.85	1.84	N.D.	N.D.	12.42	2.48	N.D.	N.D.	0.31	0.15	0.011	0.00	0.00	0.003	0.00	0.00	0.206
CL1155.Contig1	glutathione S-transferase Mu 3	3.40	0.95	N.D.	N.D.	20.55	3.13	N.D.	N.D.	0.17	0.05	0.000	0.00	0.00	0.002	0.00	0.00	0.002
Unigene5253	glutathione peroxidase	6.94	1.21	N.D.	N.D.	10.88	1.95	N.D.	N.D.	0.64	0.11	0.036	0.00	0.00	0.003	0.00	0.00	0.003
**Nervous System-Related Molecules**																		
Unigene6214	N-acetylated alpha-linked acidic dipeptidase 2	5.39	4.28	11.05	0.74	22.38	1.63	6.42	0.38	0.24	0.19	0.008	0.49	0.03	0.002	0.29	0.02	0.000
CL1083.Contig2	neuroglian	3.30	0.63	N.D.	N.D.	20.66	1.85	8.32	0.41	0.16	0.03	0.000	0.00	0.00	0.001	0.40	0.02	0.000
Unigene14124	Cysteine string protein	N.D.	N.D.	N.D.	N.D.	6.50	0.63	N.D.	N.D.	only in cyp		0.001	only in cyp		0.001	only in cyp		0.001
CL745.Contig1	Fasciclin-2	4.75	0.73	N.D.	N.D.	17.03	3.42	3.67	0.12	0.28	0.04	0.002	0.00	0.00	0.004	0.22	0.01	0.001
CL716.Contig1	Fasciclin-2	7.65	1.22	1.40	0.01	28.50	5.46	6.16	2.20	0.27	0.04	0.001	0.05	0.00	0.005	0.22	0.08	0.002
CL6416.Contig1	Acetylcholine receptor subunit alpha-type acr-16	N.D.	N.D.	4.82	0.36	7.27	0.53	N.D.	N.D.	0.00	0.00	0.000	0.66	0.04	0.008	0.00	0.00	0.000
Unigene11480	ELAV-like protein 2	N.D.	N.D.	N.D.	N.D.	9.65	3.46	N.D.	N.D.	only in cyp		0.011	only in cyp		0.011	only in cyp		0.011
**Protein Modification**																		
CL78.Contig2	cyclophilin type peptidyl-prolyl cis-trans isomeras	43.10	6.70	19.85	3.57	86.99	9.51	26.99	4.82	0.50	0.08	0.003	0.23	0.04	0.002	0.31	0.06	0.001
CL3896.Contig1	malectin-A	5.27	1.15	N.D.	N.D.	13.15	3.71	N.D.	N.D.	0.40	0.09	0.015	0.00	0.00	0.006	0.00	0.00	0.006
Unigene7031	Putative protein disulfide-isomerase A4	12.76	1.02	N.D.	N.D.	43.82	15.45	N.D.	N.D.	0.29	0.02	0.043	0.00	0.00	0.010	0.00	0.00	0.001
CL18.Contig2	Peptidyl-prolyl cis-trans isomerase	12.15	0.56	6.79	0.22	53.30	5.77	15.90	1.71	0.07	0.02	0.000	0.13	0.00	0.000	0.30	0.03	0.000
CL7127.Contig1	cyclophilin B	3.47	1.41	N.D.	N.D.	6.50	0.47	N.D.	N.D.	0.53	0.22	0.031	0.00	0.00	0.000	0.00	0.00	0.000
CL14028.Contig1	receptor accessory protein 5	N.D.	N.D.	N.D.	N.D.	6.15	0.49	N.D.	N.D.	only in cyp		0.001	only in cyp		0.001	only in cyp		0.001
**Replication, Recombination and Repair**																		
CL4480.Contig1	Replication protein A 32 kDa subunit	N.D.	N.D.	N.D.	N.D.	12.43	4.10	N.D.	N.D.	only in cyp		0.010	only in cyp		0.010	only in cyp		0.010
**Signaling Molecules**																		
CL4060.Contig1	Probable G-protein coupled receptor 158	5.55	0.80	N.D.	N.D.	11.39	0.58	3.51	1.65	0.49	0.07	0.001	0.00	0.00	0.000	0.31	0.14	0.004
CL188.Contig2	Protein phosphatase 2	2.42	1.15	N.D.	N.D.	17.05	4.88	7.71	0.67	0.14	0.07	0.003	0.00	0.00	0.007	0.45	0.04	0.018
CL7.Contig12	CUB-serine protease	N.D.	N.D.	N.D.	N.D.	7.69	2.34	N.D.	N.D.	only in cyp		0.009	only in cyp		0.009	only in cyp		0.009
CL8242.Contig1	Dipeptidyl peptidase 4	N.D.	N.D.	N.D.	N.D.	5.77	1.80	N.D.	N.D.	only in cyp		0.009	only in cyp		0.009	only in cyp		0.009
**Unclassified and Function Unkown**																		
CL62.Contig1	intracellular fatty acid-binding protein	10.22	2.47	5.56	0.21	34.45	5.10	N.D.	N.D.	0.30	0.07	0.001	0.16	0.01	0.002	0.00	0.00	0.000
CL3340.Contig1	chorion peroxidase	N.D.	N.D.	N.D.	N.D.	7.71	0.54	1.70	0.23	0.00	0.00	0.000	0.00	0.00	0.000	0.22	0.03	0.000
Unigene8333	Sterile alpha and TIR motif-containing protein	N.D.	N.D.	N.D.	N.D.	11.14	0.41	N.D.	N.D.	only in cyp		0.000	only in cyp		0.000	only in cyp		0.000
CL1705.Contig1	serine-threonine kinase receptor-associated protein	12.77	0.33	N.D.	N.D.	27.71	3.93	3.79	0.57	0.46	0.01	0.016	0.00	0.00	0.002	0.14	0.02	0.000
Unigene25709	mannosidase alpha class 2a	N.D.	N.D.	N.D.	N.D.	13.00	0.65	N.D.	N.D.	only in cyp		0.000	only in cyp		0.000	only in cyp		0.000
CL6638.Contig1	BCS-2	2.61	0.18	N.D.	N.D.	48.27	4.50	10.27	2.65	0.05	0.00	0.000	0.00	0.00	0.001	0.21	0.05	0.000
Unigene6188	CG11190 CG11190-PA	N.D.	N.D.	N.D.	N.D.	17.22	1.06	N.D.	N.D.	only in cyp		0.000	only in cyp		0.000	only in cyp		0.000
CL3280.Contig1	proprotein convertase subtilisin/kexin type 9 preproprotein	N.D.	N.D.	N.D.	N.D.	9.09	0.68	N.D.	N.D.	only in cyp		0.000	only in cyp		0.000	only in cyp		0.000
CL6163.Contig1	AGAP003142-PA	N.D.	N.D.	N.D.	N.D.	4.94	1.30	N.D.	N.D.	only in cyp		0.006	only in cyp		0.006	only in cyp		0.006
CL16604.Contig1	opsin 5	8.39	2.28	N.D.	N.D.	20.66	6.92	N.D.	N.D.	0.41	0.11	0.035	0.00	0.00	0.010	0.00	0.00	0.010
CL1826.Contig1	nodal modulator	N.D.	N.D.	N.D.	N.D.	3.66	1.40	N.D.	N.D.	only in cyp		0.011	only in cyp		0.011	only in cyp		0.011
Unigene8029	RE59172p	N.D.	N.D.	N.D.	N.D.	7.87	3.89	N.D.	N.D.	only in cyp		0.019	only in cyp		0.019	only in cyp		0.019
CL4128.Contig1	MGC84319 protein	N.D.	N.D.	N.D.	N.D.	3.77	1.92	N.D.	N.D.	only in cyp		0.020	only in cyp		0.020	only in cyp		0.020

a nau2, the stage II nauplii; nau6, the stage VI nauplii; cyp, the cyprids; juv, the juveniles; Ave, average; SD, standard deviation; N.D., not detected, *p* value was obtained by Student *t*-test.

**Table 2:** Significantly Up-regulated Proteins in Juveniles^b^.

		Protein abundance (×1000 molecules/cell)								Protein ratios								
		nau2		nau6		cyp		juv		nau2/juv			nau6/juv			cyp/juv		
Accession No.	Protein Description	ave	SD	ave	SD	ave	SD	ave	SD	ave	SD	*p*	ave	SD	*p*	ave	SD	*p*
**Structural Molecules**																		
CL8091.Contig1	calponin/transgelin	3.90	1.58	N.D.	N.D.	N.D.	N.D.	16.86	3.24	0.23	0.09	0.003	0.00	0.00	0.003	0.00	0.00	0.000
Unigene7914	AGAP007532-PA isoform 2	6.38	2.53	N.D.	N.D.	5.18	2.31	16.55	2.37	0.39	0.15	0.010	0.00	0.00	0.002	0.31	0.14	0.006
CL3067.Contig1	TPA: TPA_inf: troponin H isoform 2	6.16	1.36	N.D.	N.D.	2.64	1.10	12.09	1.37	0.51	0.11	0.007	0.00	0.00	0.001	0.22	0.09	0.001
CL400.Contig3	beta chain spectrin isoform 2	10.33	1.56	2.55	1.32	3.39	1.03	20.09	3.64	0.51	0.08	0.010	0.13	0.07	0.004	0.17	0.05	0.001
CL3753.Contig1	myosin heavy chain, non-muscle	11.78	1.10	N.D.	N.D.	1.74	0.52	25.31	2.03	0.47	0.04	0.000	0.00	0.00	0.001	0.07	0.02	0.000
CL3442.Contig1	laminin beta chain	10.59	1.84	1.53	0.73	5.66	1.45	17.23	2.33	0.61	0.11	0.017	0.09	0.04	0.001	0.33	0.08	0.002
Unigene11825	TPA: TPA_inf: troponin T isoform 1	6.52	2.55	N.D.	N.D.	5.07	1.08	29.72	1.83	0.22	0.09	0.001	0.00	0.00	0.000	0.17	0.04	0.000
CL352.Contig1	Talin-1	9.02	0.24	N.D.	N.D.	3.55	0.19	15.24	0.91	0.59	0.02	0.000	0.00	0.00	0.000	0.23	0.01	0.000
CL8008.Contig1	myosin regulatory light chain	13.53	3.34	N.D.	N.D.	N.D.	N.D.	30.63	3.10	0.44	0.11	0.004	0.00	0.00	0.001	0.00	0.00	0.001
Unigene2229	vinculin	N.D.	N.D.	N.D.	N.D.	N.D.	N.D.	21.09	2.19	only in juv		0.000	only in juv		0.001	only in juv		0.001
CL1498.Contig1	Beta-parvin	0.68	0.09	N.D.	N.D.	N.D.	N.D.	7.96	2.66	0.09	0.01	0.002	0.00	0.00	0.010	0.00	0.00	0.010
CL14241.Contig1	I-connectin	4.68	2.60	N.D.	N.D.	N.D.	N.D.	10.58	1.68	0.44	0.25	0.041	0.00	0.00	0.002	0.00	0.00	0.002
Unigene27904	titin	4.22	0.58	N.D.	N.D.	N.D.	N.D.	7.30	0.59	0.58	0.08	0.003	0.00	0.00	0.001	0.00	0.00	0.001
CL8128.Contig1	thymosin beta	N.D.	N.D.	N.D.	N.D.	N.D.	N.D.	5.23	2.60	only in juv		0.019	only in juv		0.019	only in juv		0.019
Unigene17377	Titin	N.D.	N.D.	N.D.	N.D.	N.D.	N.D.	10.82	0.12	only in juv		0.000	only in juv		0.000	only in juv		0.000
Unigene27606	Basic proline-rich protein	N.D.	N.D.	N.D.	N.D.	N.D.	N.D.	23.82	6.25	only in juv		0.006	only in juv		0.006	only in juv		0.006
Unigene28460	LOW QUALITY PROTEIN: twitchin	N.D.	N.D.	N.D.	N.D.	N.D.	N.D.	9.90	3.91	only in juv		0.012	only in juv		0.012	only in juv		0.012
**Transcription and Translation**																		
CL129.Contig2	Filamin-like	2.98	0.32	N.D.	N.D.	2.55	1.68	16.60	0.89	0.18	0.02	0.000	0.00	0.00	0.000	0.15	0.10	0.001
CL7751.Contig1	vacuolar protein sorting 35	16.07	3.96	N.D.	N.D.	9.60	5.63	30.55	7.95	0.53	0.13	0.038	0.00	0.00	0.005	0.31	0.18	0.024
CL6818.Contig1	heterogeneous nuclear ribonucleoprotein F	10.35	1.77	N.D.	N.D.	5.72	3.01	17.83	0.99	0.58	0.10	0.004	0.00	0.00	0.000	0.32	0.17	0.008
CL6551.Contig1	heterogeneous nuclear ribonucleoprotein Q	14.95	1.68	N.D.	N.D.	12.18	1.13	26.43	2.50	0.57	0.06	0.003	0.00	0.00	0.001	0.46	0.04	0.001
CL887.Contig1	ribosomal protein S6	7.82	0.16	N.D.	N.D.	8.17	1.61	24.43	3.27	0.32	0.01	0.008	0.00	0.00	0.002	0.33	0.07	0.001
CL268.Contig1	ribosomal protein S3	27.26	3.26	21.75	0.44	19.26	3.23	42.32	5.08	0.64	0.08	0.011	0.51	0.01	0.008	0.46	0.08	0.002
CL11.Contig4	Polyadenylate-binding protein 1	7.49	1.11	1.94	0.88	8.85	1.29	14.32	2.57	0.52	0.08	0.010	0.14	0.06	0.004	0.62	0.09	0.027
CL1783.Contig1	histone cluster 1, H2ad	24.18	2.70	7.38	3.48	20.69	0.71	39.18	2.48	0.62	0.07	0.002	0.19	0.09	0.002	0.53	0.02	0.000
CL6555.Contig1	Histone H1	N.D.	N.D.	N.D.	N.D.	27.55	4.67	44.74	5.73	0.00	0.00	0.001	0.00	0.00	0.001	0.61	0.10	0.016
CL6298.Contig1	Elongation factor 2	27.72	0.80	3.13	0.56	29.21	0.85	51.52	4.68	0.54	0.02	0.001	0.06	0.01	0.000	0.57	0.02	0.001
Unigene18926	DNA topoisomerase 2	N.D.	N.D.	N.D.	N.D.	N.D.	N.D.	6.58	2.50	only in juv		0.001	only in juv		0.014	only in juv		0.014
CL1214.Contig3	DNA-directed RNA polymerase II largest subunit isoform 12	N.D.	N.D.	N.D.	N.D.	N.D.	N.D.	15.44	7.46	only in juv		0.018	only in juv		0.018	only in juv		0.018
Unigene23156	mannose-binding protein	N.D.	N.D.	N.D.	N.D.	N.D.	N.D.	5.42	0.24	only in juv		0.000	only in juv		0.000	only in juv		0.000
CL7413.Contig1	trans-sialidase	N.D.	N.D.	N.D.	N.D.	N.D.	N.D.	6.41	1.90	only in juv		0.008	only in juv		0.008	only in juv		0.008
**Energy and Metabolism**																		
CL2238.Contig1	phosphate carrier protein, mitochondrial-like isoform 1	24.12	4.27	33.15	2.76	27.08	2.17	56.52	4.46	0.43	0.08	0.001	0.59	0.05	0.006	0.48	0.04	0.000
CL436.Contig4	anion exchange protein 3-like isoform 1	1.66	0.07	N.D.	N.D.	N.D.	N.D.	12.77	3.17	0.13	0.01	0.012	0.00	0.00	0.005	0.00	0.00	0.005
CL3616.Contig1	6-phosphogluconate dehydrogenase, decarboxylating	16.24	5.81	9.02	2.76	9.25	3.00	30.72	6.34	0.53	0.19	0.047	0.29	0.09	0.015	0.30	0.10	0.005
CL6215.Contig1	phosphoacetylglucosamine mutase	5.90	0.45	N.D.	N.D.	N.D.	N.D.	10.72	3.36	0.55	0.04	0.048	0.00	0.00	0.008	0.00	0.00	0.008
CL1940.Contig2	galactokinase 2	2.45	0.15	N.D.	N.D.	N.D.	N.D.	10.98	0.82	0.22	0.01	0.000	0.00	0.00	0.000	0.00	0.00	0.000
CL4661.Contig3	MOXD1-like protein 2	4.95	0.70	N.D.	N.D.	N.D.	N.D.	8.12	0.68	0.61	0.09	0.006	0.00	0.00	0.001	0.00	0.00	0.001
Unigene32056	Putative glycogen [starch] synthase	N.D.	N.D.	N.D.	N.D.	N.D.	N.D.	16.80	1.84	only in juv		0.001	only in juv		0.001	only in juv		0.001
CL10045.Contig1	dihydrolipoyllysine-residue acetyltransferase component 1 of pyruvate dehydrogenase complex, mitochondrial-lik	3.62	1.36	N.D.	N.D.	N.D.	N.D.	10.61	4.21	0.34	0.13	0.037	0.00	0.00	0.014	0.00	0.00	0.014
CL339.Contig5	ATP citrate lyase	N.D.	N.D.	N.D.	N.D.	N.D.	N.D.	17.56	10.48	only in juv		0.040	only in juv		0.040	only in juv		0.040
CL1080.Contig3	Acetyl-coenzyme A synthetase, cytoplasmic	N.D.	N.D.	N.D.	N.D.	N.D.	N.D.	7.68	3.65	only in juv		0.003	only in juv		0.022	only in juv		0.022
Unigene26389	probable 4-coumarate--CoA ligase 1	4.27	2.19	N.D.	N.D.	N.D.	N.D.	11.56	3.12	0.37	0.19	0.034	0.00	0.00	0.006	0.00	0.00	0.006
CL1.Contig245	arginine kinase	19.32	3.92	26.89	8.83	4.00	4.00	46.72	6.30	0.41	0.08	0.002	0.58	0.02	0.011	0.09	0.09	0.001
Unigene11220	Estradiol 17-beta-dehydrogenase 12	N.D.	N.D.	N.D.	N.D.	6.14	2.87	12.61	1.17	0.00	0.00	0.000	0.00	0.00	0.001	0.49	0.23	0.039
**Extracellular Matrix and Adhesion Molecules**																		
CL14344.Contig1	integrin beta-PS	N.D.	N.D.	N.D.	N.D.	N.D.	N.D.	15.10	6.78	only in juv		0.020	only in juv		0.020	only in juv		0.002
CL475.Contig2	neuroblast differentiation-associated protein AHNAK	13.63	2.17	N.D.	N.D.	3.18	1.48	35.33	0.44	0.39	0.06	0.005	0.00	0.00	0.000	0.09	0.04	0.003
Unigene6163	Basement membrane-specific heparan sulfate proteoglycan core protein	8.58	3.68	N.D.	N.D.	15.42	0.96	40.67	7.04	0.21	0.09	0.002	0.00	0.00	0.003	0.38	0.02	0.002
Unigene32829	chorion peroxidas	N.D.	N.D.	N.D.	N.D.	N.D.	N.D.	8.17	3.05	only in juv		0.011	only in juv		0.011	only in juv		0.011
Unigene9044	probable chitinase 3	N.D.	N.D.	N.D.	N.D.	N.D.	N.D.	7.82	3.57	only in juv		0.016	only in juv		0.016	only in juv		0.016
**Cell Signaling**																		
CL260.Contig1	14-3-3 zeta	41.16	3.94	18.00	5.25	34.99	3.48	72.75	2.10	0.57	0.05	0.000	0.25	0.07	0.001	0.48	0.05	0.000
CL16749.Contig1	CAMP-dependent protein kinase	5.69	1.37	N.D.	N.D.	N.D.	N.D.	11.69	3.06	0.49	0.12	0.032	0.00	0.00	0.006	0.00	0.00	0.006
**Shell Calcification**																		
CL13807.Contig1	Carbonic anhydrase 2	13.78	1.84	5.09	2.44	N.D.	N.D.	24.43	1.76	0.56	0.08	0.002	0.21	0.10	0.003	0.00	0.00	0.000
CL15286.Contig1	alpha-carbonic anhydrase	N.D.	N.D.	N.D.	N.D.	N.D.	N.D.	31.20	3.76	only in juv		0.001	only in juv		0.001	only in juv		0.001
**Protein Degradation**																		
CL7237.Contig1	Ubiquitin-like modifier-activating enzyme 1	14.57	4.98	N.D.	N.D.	N.D.	N.D.	30.21	4.63	0.48	0.16	0.021	0.00	0.00	0.002	0.00	0.00	0.000
CL6617.Contig2	26S proteasome non-ATPase regulatory subunit 2	11.37	2.49	N.D.	N.D.	9.92	0.80	18.94	2.15	0.60	0.13	0.019	0.00	0.00	0.001	0.52	0.04	0.002
**Protein Modification**																		
Unigene11234	AGAP009694-PA	14.51	0.20	5.10	1.23	6.33	2.24	22.59	1.70	0.64	0.01	0.011	0.23	0.05	0.001	0.28	0.10	0.001
CL210.Contig1	AGAP007393-PB	21.04	0.59	5.71	3.41	12.50	2.86	37.85	3.43	0.56	0.02	0.001	0.15	0.09	0.003	0.33	0.08	0.001
**Cell Differentiation**																		
CL9909.Contig1	n-myc downstream regulated	29.52	5.94	N.D.	N.D.	27.01	4.12	59.86	1.69	0.49	0.10	0.002	0.00	0.00	0.000	0.45	0.07	0.000
CL439.Contig1	kakapo	4.36	1.36	0.33	0.15	1.18	0.18	18.20	0.40	0.24	0.07	0.007	0.02	0.01	0.000	0.07	0.01	0.000
CL4061.Contig1	kakapo	6.09	0.48	N.D.	N.D.	1.07	0.07	12.33	0.36	0.49	0.04	0.000	0.00	0.00	0.000	0.09	0.01	0.000
Unigene14025	kakapo	1.46	0.16	N.D.	N.D.	N.D.	N.D.	10.26	2.18	0.14	0.02	0.001	0.00	0.00	0.004	0.00	0.00	0.004
CL3810.Contig1	kakapo	N.D.	N.D.	N.D.	N.D.	N.D.	N.D.	6.52	2.23	only in juv		0.010	only in juv		0.010	only in juv		0.010
**Cell Proliferation**																		
CL3176.Contig1	annexin 1	N.D.	N.D.	N.D.	N.D.	2.21	1.90	10.63	1.41	0.00	0.00	0.001	0.00	0.00	0.001	0.21	0.18	0.030
Unigene14024	ovulatory protein-2 precursor	N.D.	N.D.	N.D.	N.D.	N.D.	N.D.	23.76	7.63	only in juv		0.009	only in juv		0.009	only in juv		0.009
**Stress-induced Protein**																		
CL364.Contig1	Glycoprotein 93 CG5520-PA	8.11	1.68	12.89	0.41	6.12	0.64	31.49	2.17	0.26	0.05	0.000	0.41	0.01	0.001	0.19	0.02	0.000
CL405.Contig3	70kDa heat shock protein	20.10	2.71	21.29	0.08	19.17	2.38	40.46	4.34	0.50	0.07	?????	0.53	0.00	0.012	0.47	0.06	0.001
CL695.Contig1	Cct5-prov protein	6.48	0.94	2.52	0.10	3.05	0.20	13.78	1.22	0.47	0.07	0.001	0.18	0.01	0.000	0.22	0.01	0.000
**Inorganic Ion Transportaion**																		
CL3156.Contig2	Na+/K+ ATPase alpha subunit	35.37	1.81	10.17	0.91	22.21	0.32	61.04	2.01	0.58	0.03	0.000	0.17	0.01	0.000	0.36	0.01	0.000
CL12979.Contig1	Na+/K+ ATPase beta subunit	22.21	1.72	11.59	2.01	N.D.	N.D.	35.44	3.73	0.63	0.05	0.004	0.33	0.06	0.003	0.00	0.00	0.001
Unigene13663	nitric oxide synthase	N.D.	N.D.	N.D.	N.D.	N.D.	N.D.	7.09	2.68	only in juv		0.001	only in juv		0.014	only in juv		0.014
CL1831.Contig1	ribophorin I	9.08	0.45	N.D.	N.D.	N.D.	N.D.	32.37	6.47	0.28	0.01	0.013	0.00	0.00	0.003	0.00	0.00	0.003
**Cell Wall/Membrane/Envelope Biogenesis**																		
CL7195.Contig1	Vigilin	10.00	0.71	N.D.	N.D.	4.06	1.08	18.07	3.72	0.55	0.04	0.016	0.00	0.00	0.004	0.22	0.06	0.002
**Cell Cycle Control**																		
CL184.Contig1	uncharacterized protein C6orf168	16.46	1.09	8.23	0.01	10.49	2.86	32.76	2.27	0.50	0.03	0.000	0.25	0.00	0.002	0.32	0.09	0.001
**Intracellular Trafficking, Secretion, and Vesicular Transport**																		
CL1315.Contig1	clathrin heavy chain-like isoform 1	18.28	0.30	3.21	1.39	13.58	0.22	33.17	1.29	0.55	0.01	0.000	0.10	0.04	0.000	0.41	0.01	0.000
CL1268.Contig2	signal recognition particle receptor subunit alpha homolog isoform	1.70	0.51	N.D.	N.D.	N.D.	N.D.	5.16	1.36	0.33	0.10	0.009	0.00	0.00	0.005	0.00	0.00	0.005
**Unclassified and Function Unkown**																		
Unigene24129	endothelin-converting enzyme 1	3.99	0.31	N.D.	N.D.	N.D.	N.D.	20.63	5.45	0.19	0.02	0.017	0.00	0.00	0.006	0.00	0.00	0.000
Unigene29044	Nuclear receptor coactivator 5	4.10	1.55	N.D.	N.D.	N.D.	N.D.	13.59	1.78	0.30	0.11	0.003	0.00	0.00	0.001	0.00	0.00	0.000
CL3782.Contig1	sporozoite surface protein	3.16	0.65	1.15	0.01	N.D.	N.D.	7.27	2.70	0.43	0.09	0.045	0.16	0.00	0.032	0.00	0.00	0.012
CL6558.Contig1	anion exchange protein 2-like isoform 2	N.D.	N.D.	N.D.	N.D.	N.D.	N.D.	7.42	0.42	only in juv		0.000	only in juv		0.000	only in juv		0.000
CL9869.Contig1	dendritic cell protein	9.57	1.13	2.68	0.10	4.73	1.77	16.63	3.49	0.58	0.07	0.023	0.16	0.01	0.005	0.28	0.11	0.005
Unigene26106	CD109 antigen	N.D.	N.D.	N.D.	N.D.	2.14	0.26	5.40	1.06	0.00	0.00	0.003	0.00	0.00	0.003	0.40	0.05	0.003
CL7633.Contig1	Aldose reductase	15.90	2.74	3.86	1.69	16.73	1.15	30.80	3.24	0.52	0.09	0.004	0.13	0.05	0.001	0.54	0.04	0.002
CL987.Contig1	CG5149	6.16	1.60	2.24	0.56	N.D.	N.D.	12.88	4.05	0.48	0.12	0.040	0.17	0.04	0.016	0.00	0.00	0.008
CL6595.Contig1	settlement inducing protein complex	N.D.	N.D.	0.87	0.03	N.D.	N.D.	5.29	1.34	0.00	0.00	0.005	0.17	0.01	0.008	0.00	0.00	0.005
CL217.Contig3	cross-beta structure silk protein 1	N.D.	N.D.	N.D.	N.D.	N.D.	N.D.	8.58	0.80	only in juv		0.000	only in juv		0.001	only in juv		0.001
CL7617.Contig1	sodium-driven chloride bicarbonate exchanger-like isoform 3	N.D.	N.D.	N.D.	N.D.	N.D.	N.D.	5.97	1.04	only in juv		0.000	only in juv		0.003	only in juv		0.003
CL608.Contig2	trans-sialidase	2.21	1.24	N.D.	N.D.	N.D.	N.D.	4.70	0.40	0.47	0.26	0.047	0.00	0.00	0.001	0.00	0.00	0.001
CL4385.Contig1	NSFL1 cofactor p47	2.83	1.08	N.D.	N.D.	N.D.	N.D.	10.94	0.96	0.26	0.10	0.002	0.00	0.00	0.001	0.00	0.00	0.001
CL5942.Contig1	lipoprotein receptor	8.46	4.91	15.12	7.57	3.13	2.73	33.71	2.88	0.25	0.15	0.004	0.45	0.22	0.038	0.09	0.08	0.004
CL1729.Contig1	C-type lectin 4	8.03	2.13	N.D.	N.D.	N.D.	N.D.	13.94	2.22	0.58	0.15	0.032	0.00	0.00	0.002	0.00	0.00	0.002
Unigene4943	cystathionine-beta-synthase	N.D.	N.D.	N.D.	N.D.	N.D.	N.D.	5.63	0.55	only in juv		0.001	only in juv		0.001	only in juv		0.001
Unigene26120	cysteine-rich-protein	N.D.	N.D.	N.D.	N.D.	N.D.	N.D.	36.29	3.56	only in juv		0.001	only in juv		0.001	only in juv		0.001
CL2143.Contig2	cement protein-100k	N.D.	N.D.	N.D.	N.D.	N.D.	N.D.	9.77	3.13	only in juv		0.009	only in juv		0.009	only in juv		0.009
CL1152.Contig1	BCS-4	N.D.	N.D.	N.D.	N.D.	N.D.	N.D.	35.37	4.58	only in juv		0.001	only in juv		0.001	only in juv		0.001
Unigene7077	transglutaminase	N.D.	N.D.	N.D.	N.D.	N.D.	N.D.	8.11	4.26	only in juv		0.021	only in juv		0.021	only in juv		0.021

b nau2, the stage II nauplii; nau6, the stage VI nauplii; cyp, the cyprids; juv, the juveniles; Ave, average; SD, standard deviation; N.D., not detected, *p* value was obtained by Student *t*-test.

**Figure 3:**
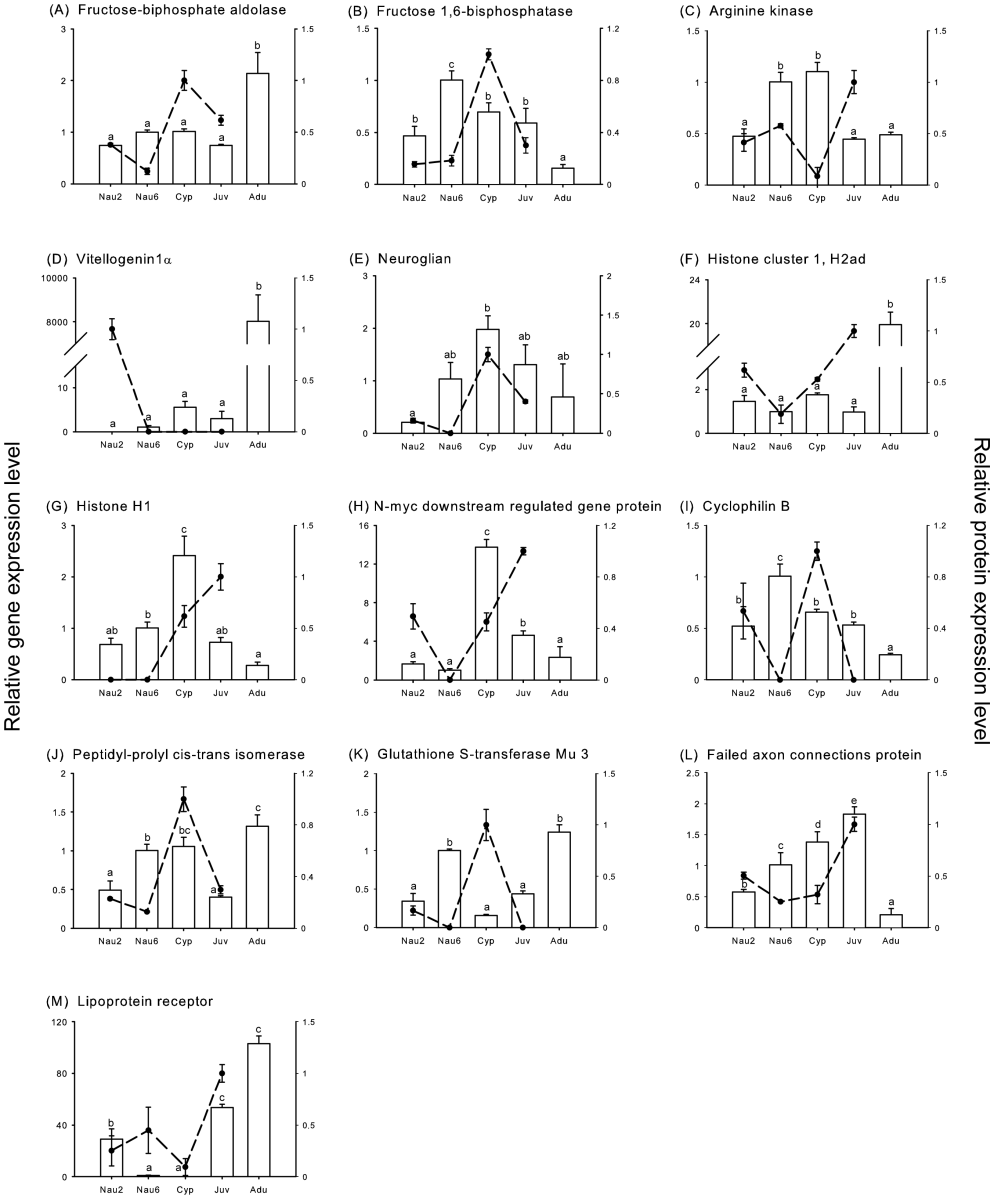
Quantitative real-time PCR (qRT-PCR) results of the levels of 13 genes with protein products that were significantly up-regulated in either cyprids or juveniles (white bars). The corresponding protein expression levels are presented as line charts. The detected developmental stages included stage II nauplii (Nau2), stage VI nauplii (Nau6), cyprids (Cyp), young juveniles (Juv) and adults (Adu). Values are expressed as the mean ± SD of 3 different experimental replicates. In the figures, a, b, c, d and e in the figures showed significantly different expression patterns among the samples detected, as determined by one-way ANOVA followed by Tukey's post-hoc test (*P*<0.05).

To analyze the gene expression of the selected proteins, qRT-PCR was conducted. As shown in Figure 3, a positive correlation between transcription and translation expression patterns was detected in several proteins, such as neuroglian, failed axon connection protein and lipoprotein receptor. In contrast, the expression trends observed for some proteins, including fructose-bisphosphate aldolase, histone cluster 1 H2ad and glutathione S-transferase Mu 3, did not display any similarity with their gene expression profiles. Interestingly, some transcripts accumulated prior to the peak expression of the protein products, as observed for fructose 1,6-bisphosphatase, arginine kinase, histone H1, n-myc downstream gene protein, cyclophilin B and peptidyl-prolyl cis-trans isomerase.

## Discussion

Barnacle larval settlement includes the attainment of competency by swimming cyprids, attachment to the substratum, and the metamorphosis of attached cyprids into juveniles, which involve energy-related, neurotransmission-related, and cell proliferation and differentiation-related molecules are involved [5]. With a special focus on larval settlement and subsequent juvenile development, we identified 80 and 95 proteins that were significantly up-regulated in the cyprid and the juvenile proteome, respectively.

### Proteins that were significantly up-regulated in cyprids

#### Energy and metabolism proteins

Twenty-four energy-related proteins were significantly up-regulated in cyprids, accounting for approximately 30% of the up-regulated proteins in the larval stage (shown in Table 1). Fructose-bisphosphate aldolase, fructose 1,6-bisphosphatase, glucose-6-phosphate isomerase, glucosamine-6-phosphate isomerase 1 and mannose-6-phosphate isomerase are involved in the metabolism of fructose 6-phosphate, which is one of the key products in glycolysis and its reverse process, gluconeogenesis, for converting a non-carbohydrate to glucose [27]. Because cyprids do not feed, they rely on stored lipids and proteins as primary energy sources to swim, search for a suitable surface for attachment and metamorphosis [28]. The up-regulation of fructose 1,6-bisphosphatase in cyprids suggested that lipids and proteins were converted to glucose via active gluconeogenesis to support cyprid energy consumption. Interestingly, the transcript displayed a higher expression level in stage VI nauplii than in other stages (Figure 3B). Furthermore, because two of the enzymes catalyzed reactions in both glycolysis and gluconeogenesis, fructose-bisphosphate aldolase and glucose-6-phosphate isomerase might play important roles in maintaining the balance between these two metabolic pathways in cyprids.

In the present study, acetyl-CoA acetyltransferase was significantly up-regulated during the cyprid stage, which is consistent to the results of an early study demonstrating that the acetyl-CoA acetyltransferase gene was significantly up-regulated during this stage [29]. Acetyl-CoA carboxylase 2 and acyl-CoA oxidase participate in the catabolism of lipids through fatty acid oxidation [30], [31]. The product of acetyl-CoA enters citric acid cycle to produce energy. Acetyl-CoA acetyltransferase is involved in several metabolic pathways, including ketone body synthesis and degradation, fatty acid metabolism and pyruvate metabolism. Cyprids become less active and their settlement rate decreases in response to treatment with butenolide [32], as acetyl-CoA acetyltransferase is one of the binding targets of butenolide in barnacle cyprids and is involved in the inhibition of larval settlement triggered by butenolide [29]. Taken together, these results indicated that acetyl-CoA acetyltransferase is a crucial enzyme for maintaining barnacle cyprid activity and their subsequent successful settlement.

**Figure 4:**
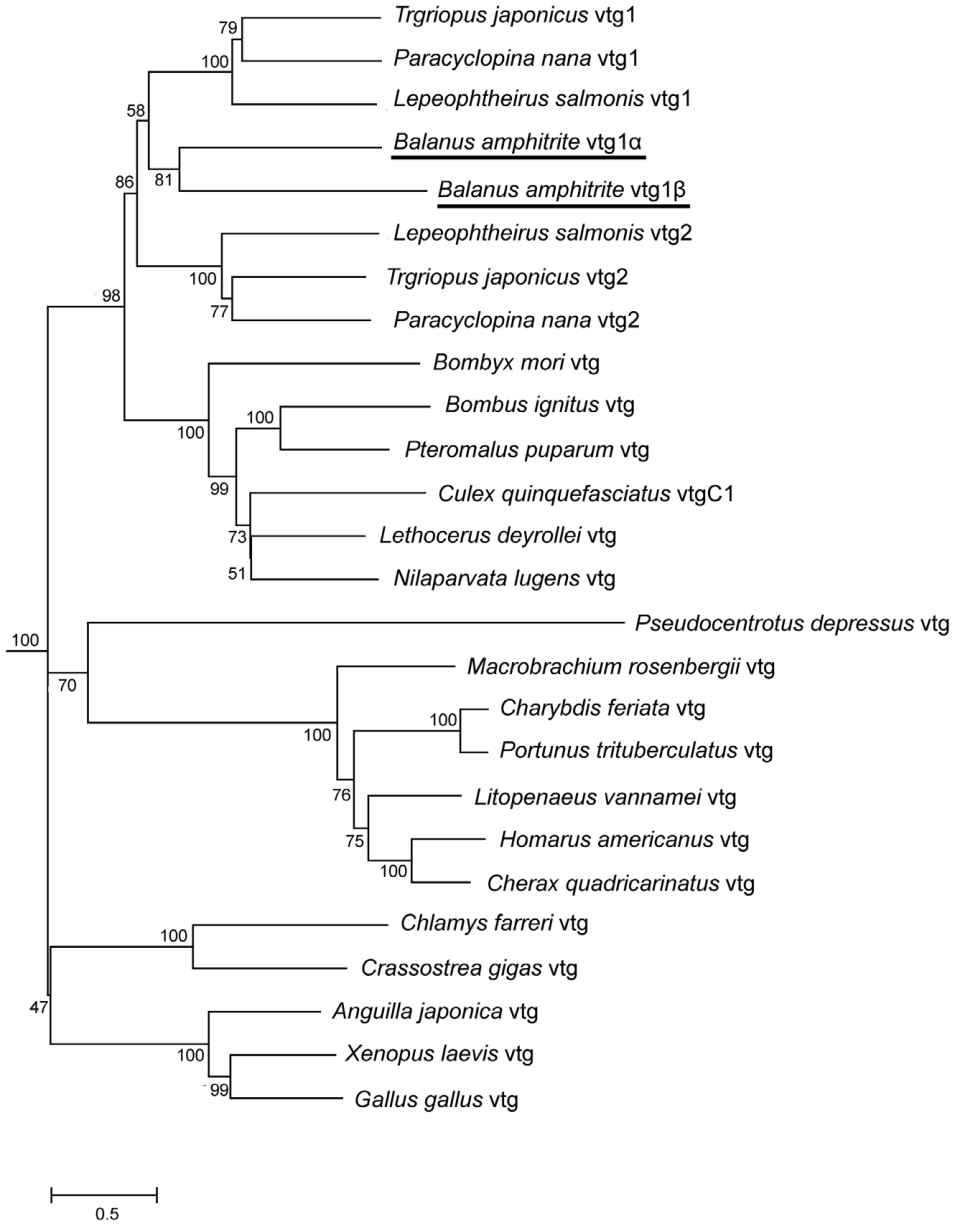
Neighbor-joining tree based on vitellogenin protein sequences. Branch lengths represent substitutions per site, and numbers at each node represent bootstrap values. The sequences used were as follows: *Tigriopus japonicas* vtg1 (ABZ91537), *Tigriopus japonicas* vtg2 (ACJ12892), *Paracyclopina nana* vtg1 (ADD73551), *Paracyclopina nana* vtg2 (ADD73552), *Lepeophtheirus salmonis* vtg1 (ABU41134), *Lepeophtheirus salmonis* vtg2 (ABU41135), *Bombyx mori* vtg (BAA06397), *Bombus ignites* vtg (ACM46019), *Pteromalus puparum* vtg (ABO70318), *Culex quinquefasciatus* vtg (AAV31930), *Lethocerus deyrollei* vtg (BAG12118), *Nilaparvata lugens* vtg (AEL22916), *Pseudocentrotus depressus* vtg (AAK57983), *Macrobrachium rosenbergii* vtg (BAB69831), *Charybdis feriata* vtg (AAU93694), *Portunus trituberculatus* vtg (AAX94762), *Litopenaeus vannamei* vtg (AAP76571), *Homarus americanus* vtg (ABO09863), *Cherax quadricarinatus* vtg (AAG17936), *Chlamys farreri* vtg (ADE05540), *Crassostrea gigas* vtg (BAC22716), *Anguilla japonica* vtg (AAV48826), *Gallus gallus* vtg (AAA49139.1), and *Xenopus laevis* vtg (AAA49982).

In the barnacle proteome, two vitellogenins were identified. Vitellogenin 1α was only detected in the stage II nauplii (Table S2); however, its gene expression level was 1,000 times higher in adults (Figure 3D), suggesting that this maternal vitellogenin 1α might function as a storage protein for embryonic and early larval development, similar to the general vitellogenin in other species. Interestingly, unlike vitellogenin 1α, vitellogenin 1β was highly expressed in stage VI nauplii and cyprids (Table S2), and its expression pattern was similar to that of the cyprid major protein. Cyprid major protein is utilized as an energy resource during cyprid settlment and metamorphosis [33]. It was identified as a vitellogenin 1β-like protein based on the results of SDS-PAGE and LC-MS analysis [34]. To better explore the relationship between the two vitellogenins detected in *B. amphitrite*, we conducted phylogenetic reconstruction (Figure 4). Our results revealed the monophyly of copepod Vitellogenin 1 (BS  = 100), copepod Vitellogenin 2 (BS  = 100) and the sister-relationship between Vitellogenin 1α and 1β (BS  = 81). Furthermore, the last two genes also formed the sister taxon of copepod Vitellogenin 1 (BS  = 58). These relationships suggest that barnacle Vitellogenins have functions similar to other vitellogenins; however, Vitellogenin 1β has evolved a novel and related function after gene duplication such that it is highly expressed in stage VI nauplii and cyprids rather than embryos or adults.

#### Nervous system-related molecules

Cyprids possess a more complicated nervous system compared with nauplii and adults [35]. Extensive studies have been conducted to investigate neurotransmitters such as serotonin and prostanoid [36], [37]. In the current study, we detected one neurotransmitter receptor, ie, acetylcholine receptor, with a high expression level in cyprids. This receptor binds to the neurotransmitter acetylcholine, which is involved in barnacle cyprid muscular contraction and cement gland exocytosis [38]. Elevated levels of acetylcholine lead to a higher settlement rate of cyprids [38]. The up-regulation of this receptor in cyprids herein confirmed that acetylcholine has a key role in barnacle larval settlement.

N-acetylated alpha-linked acidic dipeptidase is responsible for the cleavage of the neuropeptide N-acetyl-L-aspartate-L-glutamate (NAAG), which is abundant in the central nervous system and functions as a neurotransmitter [39]. A previous study revealed the effects of NAAG on the reduction of cAMP levels and the release of GABA [40], [41]. In *B. amphitrite*, the intracellular cAMP level affects the patterns of cyprid settlement [42]. As a neurotransmitter, GABA controls thoracic muscle contraction and modulates eye vision and antennule activity [43]. Thus, in cyprids, N-acetylated alpha-linked acidic dipeptidase may be involved in regulating of cAMP and GABA levels by catabolizing NAAG.

In addition, we identified neuroglian, which belongs to the Ig superfamily and is essential for the development of neuroglia and the formation of synapses [44]. Neuroglian gene expression increased during larval development and peaked in cyprids, but no significant difference was observed after settlement (Figure 3E). The expression of its protein product displayed similar trends during all larval stages but decreased after settlement (Table 1). Barnacle neuroglian was found to contain 6 Ig superfamily domains and possess region homologies to cell adhesion molecules or a neuroglian-like isoform in insects. It has been reported that neuroglian regulates glial morphogenesis and antennal lobe development in *Drosophila* larvae undergoing metamorphosis [45].

#### Signaling molecules

In the present study, the CUB-serine protease was found to be uniquely expressed in cyprids, the antennules of which contain an olfactory receptor neuron-like structure. The CUB-serine protease was first located in the olfactory organ and eyestalk of the spiny lobster *Panulirus argus*, which suggested that it functions in the olfactory system [46]. When barnacle cyprids were treated with the anti-settlement compound meleagrin, the protein expression of the CUB-serine protease was modulated [34]. Up-regulation of the CUB-serine protease in barnacle cyprids suggested that this protein might be involved in the cyprid olfactory chemoreception system during the search for an ideal settlement spot.

Furthermore, in the present study, we identified a putative G protein-coupled receptor in the barnacle proteome that was significantly up-regulated during the cyprid stage. In the red abalone *Haliotis refescens*, a G protein-coupled receptor and downstream PKA-dependent cyclic AMP (cAMP) pathway are crucial participants in the pathway controlling larval settlement and metamorphosis [47]. In *B. amphitrite*, it has been suggested that a G protein-coupled receptor binds to the exogenous metamorphic cue while the settlement signal is conducted via a PKA independent pathway [48], [49]. Therefore, the G protein-coupled receptor identified in the current study may play a crucial role in recognizing of the settlement cue. Interestingly, we did not find any other cAMP-pathway-related proteins that were differentially expressed in cyprids, yet 1 cAMP-dependent protein kinase (PKA) was up-regulated in juveniles. Signal transduction regulated by cAMP is required for the development of a metazoan [50]. Down-regulation of PKA in cyprids and up-regulation in juveniles indicates that this PKA may be important for juvenile development rather than for larval settlement.

#### Structural proteins

7 structural proteins were differentially expressed during the cyprid stage. Among them, 5 belonged to the connectin/titin family, including projectin, twitchin and 3 titins (Table 1). Titins are giant proteins that contribute to muscle assembly and contraction, especially in striated muscle [51]. In barnacle cyprids, striated muscle is distributed in antennules, thoracopods and their related muscles [52]. Antennules and thoracopods function differently in cyprids: antennules are the prime locomotor and sensory apparatus, which helps these organisms contact with the substratum during the attachment process. In contrast, thoracopods enable cyprids to move or stay in the water column [53]. The up-regulation of the proteins involved in the assembly and contraction of striated muscle supported the active searching and swimming behavior displayed during barnacle larval settlement.

#### Functionally ungrouped proteins

In addition to the functional groups mentioned above, we also identified many proteins that belonged to some other functional groups or which unknown functions. Opsin 5 showed the most similarity to *Limulus* opsin 5, the protein component of visual pigment that is sensitive to visible light (400–700 nm) [54]. Barnacle cyprids, which have a pair of stage-specific compound eyes and 1 naupliar eye, display a phototactic response to light flux. The photoreception in cyprids has been suggested to be associated with a visual sensory system needed for larval settlement and distribution [55].

A previous study described the cloning of 6 barnacle cyprids larva-specific genes (*bcs*) by screening cDNA libraries [8]. As an ‘early’ gene, *bcs-2* was highly expressed in cyprids, and then decreased dramatically when larval settlement occurred [8]. In the present study, the protein product of this gene displayed the same abundance in cyprids but could not be detected in juveniles, which suggested a specific role for Bcs-2 in barnacle cyprids.

### Proteins that were significantly up-regulated in juveniles

#### Energy and metabolism proteins

In juveniles, we identified 12 proteins that were related to energy and metabolism. Among these 12 proteins, arginine kinase was significantly up-regulated during the juvenile stage, whereas its gene expression was 2-fold higher in stage VI nauplii and cyprids than in the other 3 stages (Figure 3C). These results indicated that the arginine kinase transcript might be accumulated prior to that of fructose 1,6-bisphosphatase. Arginine kinase often serves as a temporal energy buffer system, increasing the efficiency of the reversible phosphorylation of arginine by ATP and of fluctuating energy requirements in invertebrates [56], [57]. In addition, both pyruvate dehydrogenase and ATP citrate lyase were up-regulated in juveniles. This up-regulation might ensure the production of acetyl-CoA for subsequent energy production and fatty acid synthesis, as the pyruvate dehydrogenase complex catalyzes the transformation from pyruvate to acetyl-CoA via pyruvate decarboxylation. Alternately, this phenomenon might stimulate the generation of acetyl-CoA from acetate and CoA via the catalysis of acetyl-CoA synthetase [58]. In contrast, ATP citrate lyase is involved in the generation of acetyl CoA together with the ATP hydrolysis [59].

#### Structural proteins

In the juvenile proteome, 17 structural proteins were significantly up-regulated. Among them, several proteins were found to be involved in muscle assembly and contraction, including 4 connectin/titin family proteins, 2 troponin isoforms, myosin light chain and transgelin (Table 2). In addition, several actin-binding proteins were detected, such as vinculin, laminin, talin, β-parvin, and thymosin β. Spectrin is another actin scaffold protein that helps maintain the shape of the cell and plays a crucial role in the survival and development of *Drosophila* larval [60]. The up-regulation of these cytoskeletal proteins was not unexpected, as barnacles must undergo drastic tissue degeneration and organ remodeling during metamorphosis, such as the reduction of larval muscles, the formation of juvenile muscles, rotation of the thorax, the development of cirri, and raising of the body, among others [61].

#### Proteins related to transcription and translation

Thirteen proteins related to transcription and translation were significantly up-regulated during the juvenile stage. By comparison, only 6 proteins were up-regulated during the cyprid stage. We identified two heterogeneous nuclear ribonucleoproteins (F and Q) and 1 polyadenylate-binding protein, all of which were found to be involved in gene transcription and post-transcriptional modification by regulating mRNA metabolism [62], [63]. A similar expression pattern was observed for histone H2ad and histone H1, which are coupled to DNA replication, and elongation factor 2, which facilitates translation elongation. Interestingly, the gene expression of histone H2ad did not show much fluctuation during the larval stage or in juveniles but increased dramatically in adults. However, the histone H1 gene was expressed at least 2.4-times higher in cyprids than during any other stage (Figures 3F & 3G). These results suggested that histone H1 might be important for juvenile development only and that histone H2ad might function in both juveniles and adults. In short, the up-regulation of these proteins indicated that active transcription and translation might occur in barnacle juveniles.

#### Proteins related to cell proliferation and differentiation

Proteins involved in cell proliferation and differentiation were significantly up-regulated in juveniles only. In total, we identified 4 kakapos, one of which was detected only in juveniles. Kakapo, a cytoskeletal-associated protein expressed in *Drosophila* tendon cells, may be important for muscle-dependent tendon cell differentiation [64]. In barnacles, tendon cells, which are specially organized epithelial cells, function as a link between muscles and the overlying chitinous exoskeleton [65]. Up-regulation of these 4 kakapo proteins indicated that they have crucial functions in cell differentiation, mainly in the barnacle integument. In addition, N-myc downstream regulated gene (NDRG) protein was highly abundant in the juvenile proteome. This protein was down-regulated during the larval stage and increased in juveniles (Table 2); however, its gene expression peaked in cyprids (Figure 3H). The NDRG protein family contributes to cell differentiation and proliferation in diverse tissues of various animals [66]. Our results suggested that NDRG functions in cell differentiation processes associated with tissue reorganization in barnacle juveniles.

In the current study, we identified one annexin that was differentially expressed in juveniles. The expression of this protein was first detected in cyprids and then peaked in juveniles (4.8-fold higher than in cyprids) (Table 2). Annexins comprise a group of proteins that bind to phospholipids in a calcium-dependent manner. Previous studies have shown that the expression patterns of annexins change significantly when cells undergo proliferation or differentiation [67]. During silkworm metamorphosis of *Bombyx mori*, 20-hydroxyecdysone (20-HE) triggers programmed cell death to remove larval-specific tissues. Annexin, identified as a 20-HE inducible gene, was shown to be involved in this process [68]. The 20-HE gene has also been detected in barnacle cyprids, and its regulation of barnacle larval metamorphosis has been verified [69]. Although the detailed mechanism by which 20-HE affects barnacle larval metamorphosis remains unclear, the expression levels of annexin might be modulated by the tissue degeneration reorganization that occurs during barnacle larval development and metamorphosis, which in turn might be regulated by 20-HE.

#### Shell calcification proteins

Two carbonic anhydrases related to shell calcification were significantly up-regulated in juveniles and might be involved in biomineralization in *B. amphitrite*, especially α CA, which was uniquely expressed in juveniles. Carbonic anhydrase (CA) participates in a variety of metabolic pathways and is widely distributed in the tissues that are responsible for the formation of calcium carbonate [70]. It is also important for the molting cycle in crustaceans [71]. Inhibition of CA activity prevents shell development and growth of the barnacle *Balanus improvisus*, resulting in a failure in the initiation of normal development [70].

#### Functionally ungrouped proteins

In the present study, we discovered that the protein product of the *bcs-4* gene was uniquely expressed in juveniles. Compared with *bcs-2*, *bcs-4* is a ‘late’ gene showing weak expression levels in young cyprids that have recently molted from nauplii, increasing gradually as cyprids age, and beginning to decline in settled larvae [8]. This time lag between the expression of gene and protein supports the possibility that accumulation of the gene is required prior to protein function in juveniles.

In addition to Bsc-4, one cement protein-100k was found to be uniquely expressed during the juvenile stage. Its gene transcript was detected in the adult but not in the larval transcriptome [5]. In fact, several proteins have been identified, such as two 20 kDa-cement proteins characterized in the cyprids of *B. amphitrite* and large cement proteins fractionated from the adult cement of *Megabalanus rosa* (100 kDa, 68 kDa & 52 kDa) [72], [73].

Three proteins belonging to the heat shock protein (HSP) family were significantly up-regulated in juveniles. HSPs were initially known as stress-responsive proteins and later found to interface with various developmental pathways, as molecular chaperones associated with protein folding, assembly and transport [74], [75]. HSP70 is developmentally regulated in a diverse range of organisms, and HSP90 functions as a regulator during metamorphosis and the molt cycle of invertebrates [76]–[78]. Additionally, the expression levels of HSP70 and HSP90 increase in response to cell differentiation and tissue morphogenesis occur in the vetigastropod *Haliotis asinina* [79].

### Comparison of proteome profiles with other marine invertebrates

In addition to the present study, proteomic approaches have been applied to study larval attachment and metamorphosis in several marine invertebrates. Although the species were from several evolutionarily distant phyla, common changes in the proteome were observed. First, during attachment and metamorphosis, a larva goes through serial body reconstructions, including the degeneration of larval structures and the subsequent emergence of juvenile tissues. The larvae of the bryozoan *Bugula neritina* and the polychaete *Hydroides elegans*, swim via beating cilia, and all of these ciliated tissues degenerate during metamorphosis, resulting in drastic changes in structural proteins, such as actin and tubulin, during larval settlement [20], [80]. Barnacle larvae swim by beating thoracopods driven by striated muscles. Actin and tubulin were not differentially expressed in the barnacle proteomes, but several proteins related to muscle structures and muscle contraction were up-regulated. In addition to structural proteins, in the present study, proteins involved in transcription and translation, such as ribosomal proteins, histones and elongation factors, were highly expressed during larval settlement in the bryozoan *B. neritina* [20], the polychaete *Pseudopolydara vexillosa* [81] and so as in *B. amphitrite*. These findings indicated that transcription and translation level were active when larvae underwent metamorphosis.

Larval settlement is an extremely energy-consuming process, especially for the whole larval settlement process. Proteins involved in the citric acid cycle, glycolysis, and fatty acid metabolism have been found to be up-regulated during the competent larval stage of *B. neritina*, *P. vexillosa* and the polychaete *Capitella* sp. I [20], [80], [81]. Similarly, these proteins were found to be up-regulated in barnacle cyprids herein. In addition, some proteins related to energy and metabolism were also up-regulated in barnacle juveniles, as described for *P. vexillosa* [81].These findings indicated that these organisms might require different energy metabolism pathways for larval competency and metamorphosis. Interestingly, vitellogenins have been described in competent larvae of *B. neritina*, *B. anphitrite* and *Capitalla* [20], [82], indicating that the non-feeding larvae of different phyla all consume vitellogenins as an energy source.

Carbonic anhydrase was detected in juveniles of both *B. neritina* and *B. anphitrite*, which require a calcified body wall or a calcareous shell [83]. This result indicated that carbonic anhydrase is important for the calcification of marine invertebrates.

## Conclusions

This study investigated the regulation of protein expression patterns in response to larval development and settlement of the barnacle *B. amphitrite*. The utilization of label-free quantitative proteomics allowed us to conduct a comparative proteomics analysis among different developmental stages and to identify protein candidates that might be involved in barnacle larval settlement. Functional analysis of 4 proteins revealed their significant up-regulation of proteins involved in energy and metabolism, the nervous system, and signaling transduction in the cyprid stage. In addition, proteins related to cytoskeletal remodeling, translation and transcription, cell proliferation and differentiation, and biomineralization were up-regulated in the juvenile proteome. Notably, the expression patterns of some proteins, such as neuroglian, failed axon connection protein, and lipoprotein receptor, displayed the same trends as their transcripts; In contrast, the expression of fructose-1, 6-bisphosphatase, cyclophilin B, NDRG, and histone H1 transcripts occurred 1 developmental stage earlier than their protein products. The expression levels of some other proteins, such as fructose-biphosphate aldolase, were not associated with their mRNA expression. These results provide information about the molecular activities related to the changes in morphology, physiology, structure, and function that occur during barnacle larval settlement and juvenile development. Additional functional assays and characterization of protein candidates, for instance, nervous system-related proteins, signaling molecules, shell calcification proteins and heat shock proteins, could help identify more detailed molecular mechanisms underlying barnacle larval settlement.

## Supporting Information

Table S1 **Primers for genes under real-time PCR assay.**
(XLSX)

Table S2 **Total proteins identified and quantified from each LC-MS experiments.**
(XLSX)

Table S3 **Significantly up-regulated proteins in stage II nauplii.**
(XLSX)

Table S4 **Significantly up-regulated proteins in stage VI nauplii.**
(XLSX)

Table S5 **Significantly down-regulated proteins in stage II nauplii.**
(XLSX)

Table S6 **Significantly down-regulated proteins in stage VI nauplii.**
(XLSX)

Table S7 **Significantly down-regulated proteins in cyprids.**
(XLSX)

Table S8 **Significantly down-regulated proteins in juveniles.**
(XLSX)
